# Simultaneous abdominal wall defect repair and Tenckhoff catheter placement in candidates for peritoneal dialysis

**DOI:** 10.1007/s40620-015-0251-8

**Published:** 2015-11-30

**Authors:** Maurizio Sodo, Umberto Bracale, Gennaro Argentino, Giovanni Merola, Roberta Russo, Giuseppina Sannino, Tania Strazzullo, Domenico Russo

**Affiliations:** 1 General Surgical Unit, Department of Public Health, University FEDERICO II Naples, Naples, Italy; 2 Nephrology, Department of Public Health, University FEDERICO II Naples, Naples, Italy

**Keywords:** Abdominal wall defect, Peritoneal dialysis, Inguinal hernia, Peritoneal catheter

## Abstract

**Introduction:**

The presence of pre-existing abdominal wall defect (AWD) could represent a potential contraindication for peritoneal dialysis (PD) treatment. We report the results of our 6-year experience involving simultaneous repair of pre-existing AWD and catheter insertion for PD.

**Methods:**

Patients with estimated glomerular filtration rate (e-GFR) 7–10 ml/min attending a single nephrology clinic between January 2008 and December 2014 were evaluated. Simultaneous AWD repair and catheter placement was performed. For inguinal (IH) or umbilical hernia (UH), a prolene mesh repair technique was adopted. Except for one case of total anaesthesia, the surgical procedure was performed under either spinal or local anaesthesia. Ceftazidime alone or in association with quinolones was administered 1 h before surgery in a single dose. Patients were discharged 2 days after surgery, and returned to the clinic twice during the 1st week for peritoneum washing (first volume of peritoneal dialysis solution: 300 ml). From week 3, volume (2000 ml) and dwells were personalized according to the patient’s clinical condition; options were: incremental PD, standard PD, or continuous cycling PD. Surgical follow-up was planned at 1, 6, and 12 months.

**Results:**

Peritoneal catheters were inserted in 170 patients. IH, UH and incisional hernia were found in 18, 2 and 1 patients, respectively. IH was bilateral in 4 patients; concomitant IH and UH occurred in 1 patient. There were no deaths, nor intra-operative complications apart from scrotal haematoma in 1 patient. Over a mean follow-up of 551 days (range 342–1274) no hernia recurrence was registered and the peritoneal catheter continued functioning without problems.

**Conclusions:**

Simultaneous AWD repair and peritoneal catheter placement seems a reliable and safe surgical procedure that allows patients with AWD to benefit from PD treatment.

## Introduction

In patients on peritoneal dialysis (PD), the intra-abdominal pressure (IP) increases due to the flow of dialysis fluid into the peritoneal cavity. The increase of IP is proportional to the quantity of liquid introduced [1–3], and is frequently the cause of hernia. However, even a normal IP pressure may be dangerous for the abdominal wall in patients with increased body mass index, polycystic kidney disease, in those who engage in certain types of physical activity, as well as in multiparous women [1–5]. Therefore, PD is regarded as the primary cause of occurrence of abdominal wall defect (AWD) and, on the other hand, the presence of pre-existing AWD is considered a potential contraindication for PD [1–3]. However, the latter limitation is debated. To help clarify this issue, we report the results of our 6-year experience involving simultaneous repair of pre-existing AWD and catheter insertion for PD.

## Materials and methods

Patients attending a single nephrology clinic between January 2008 and December 2014 were evaluated. Patients with estimated glomerular filtration rate (e-GFR) between 7 and 10 ml/min underwent physical examination by a dedicated team of nephrologists, surgeons and skilled nurses. AWD such as inguinal, umbilical and incisional hernia were carefully checked for by surgeons. In the presence of AWD, simultaneous repair of it and peritoneal catheter placement was performed in a one-stage procedure. AWD repair preceded peritoneal catheter insertion. The surgical procedure was performed under either spinal or local anaesthesia.

In cases of inguinal hernia, the modified Lichtenstein technique was adopted [6, 7]. In brief, patients underwent tension-free hernioplasty. The inguinal canal was prepared and the hernial sac managed according to the Lichtenstein technique. The ilioinguinal nerve, iliohypogastric nerve and genital branch of the genito-femoral nerve were prepared and preserved. A semi-absorbable lightweight prolene mesh 10 × 6 cm (ULTRAPRO^®^, Ethicon Products, Somerville, NJ, USA) was placed on the inguinal floor, overlying the pubic tubercle by 2 cm, and fixed with a non-absorbable suture. After repositioning the external oblique muscle and Scarpa’s fascia, the skin was closed with a non-absorbable continuous suture. In the case of umbilical hernia the procedure was conducted according to the technique proposed by Stabilini [8].

The peritoneal catheter was inserted through longitudinal incision 2–3 cm below the umbilical transversal line. The catheter tip was located in the Douglas root. The proximal cuff was fixed to the peritoneum with an interrupted absorbable suture. The fascia was closed with an absorbable suture. The distal cuff was tied to the anterior face of the rectum muscle fascia. The catheter skin exit was directed downwards or laterally. The catheter was flushed with 20 ml of normal saline to ensure patency and correct functioning. The skin was closed with a non absorbable continuous suture.

Ceftazidime alone or in association with quinolones was administered as a single dose 1 h before surgery.

Patients were discharged 2 days after surgery, and returned to the Nephrology clinic twice during the first week for peritoneum washing (mean initial dialysis solution: 300 ml). The volume of washing solution was progressively increased during the following 3 weeks (from 1000 to 1500 to 2000 ml at weeks 1, 2, and 3, respectively). From week 3, volume (2000 ml) and dwells were personalized according to the patient’s clinical condition; options were: incremental PD, standard PD, or continuous cycling PD (CCPD). Surgical follow-up was planned at 1, 6, and 12 months. Informed consent was obtained from all participants in the study.

## Results

During the study period, peritoneal catheters were placed in 170 patients (94 males and 76 females). Among these patients, inguinal hernia, umbilical hernia and incisional hernia were found in 18, 2 and 1 patients, respectively. Inguinal hernia was bilateral in 4 patients (3 males; 1 female); concomitant inguinal hernia and umbilical hernia occurred in 1 patient. Clinical characteristics of patients with AWD are shown in Table 1. Mean age was 61 ± 11 years (range 35–80); 50 % were aged <65 years. Mean body mass index was 24.7 ± 2.6; 6 patients were over-weight, and the remaining normal weight. Diabetes was present in 6 patients.

**Table 1:** Clinical characteristics of patients

Patients	Sex	Age (years)	BMI	Cause of CRF	Hernia	Complications	Anaesthesia
1	M	55	27.4	Glomerulonephritis	IMH	–	Spinal
2	M	67	24.7	Glomerulonephritis	IBH	–	Spinal
3	M	71	29.2	Diabetes mellitus	IBH	–	Spinal
4	F	67	28.7	Diabetes mellitus	IMH	–	Spinal
5	M	66	23	Glomerulonephritis	IBH	–	Spinal
6	F	80	22.5	Diabetes mellitus	IMH/NR	–	Local
7	F	72	28.2	Diabetes mellitus	IMH	–	Spinal
8	M	68	21	Glomerulonephritis	IMH	Scrotal haematoma	Spinal
9	M	64	22.5	Glomerulonephritis	IMH	–	Spinal
10	M	70	27.6	Diabetes mellitus	IMH	–	Local
11	F	59	23.6	Glomerulonephritis	IMH	Exit site infection	Spinal
12	F	46	22.3	Glomerulonephritis	IBH	–	Spinal
13	M	56	21.8	Glomerulonephritis	IMH	–	Spinal
14	M	35	23.4	Glomerulonephritis	IMH	–	Spinal
15	M	48	22.6	Glomerulonephritis	IMH	–	Spinal
16	F	63	25.4	Glomerulonephritis	IMH	–	Spinal
17	F	54	24.9	Glomerulonephritis	IMH	–	Spinal
18	F	68	28.4	Diabetes mellitus	IMH	–	Spinal
19	F	67	22.3	Glomerulonephritis	UH	–	Spinal
20	M	44	23.4	Glomerulonephritis	INH	–	Total
21	F	61	25.4	Glomerulonephritis	UH	–	Spinal

*BMI* body mass index, *CRF* chronic renal failure, *IMH* inguinal monolateral hernia, *IBH* inguinal bilateral hernia, *NR* non-reducible, *UH* umbilical hernia, *INH* incisional hernia

The mean operative time was 55 min (range 40–130). There were no deaths, nor intra-operative complications apart from scrotal haematoma in 1 patient who was conservatively managed and recovered within 1 month. During a mean follow-up of 551 days (range 342–1274) no hernia recurrence was registered and the peritoneal catheter continued to function without any problems.

## Discussion

In our cohort of 170 patients who had been admitted to a single nephrology Unit for initiation of PD, the rate of occurrence of AWD was 15 %. Inguinal hernia was the most common AWD, being found in 13 % of patients. This incidence is similar to that reported elsewhere [9, 10] while the incidence of umbilical hernia was lower than in a previous report (>60 %) [10]; the higher incidence in that case could be due to the fact that many of those patients were obese. It is commonly thought that AWD is more common in older people. Of note, we found AWDs in some of our younger patients. This finding is in line with other reports where AWDs were found in PD patients younger that those recruited in the present study [5, 9, 10].

The results of this study are clinically relevant. They suggest that simultaneous AWD repair and peritoneal catheter placement is, on the one hand, a reliable surgical procedure and, on the other hand, that it may represent a valid option for critical patients. Indeed, the peritoneal catheter continued to function efficiently and no recurrence of AWD was registered during the long follow-up of our study. These findings suggest that repair of pre-existing AWD does not interfere with endurance of the peritoneal catheter and does not affect dialysis efficacy. It is interesting that no recurrence of AWD was registered in our patients during PD treatment. Recurrence of AWD has been related to uraemia-dependent muscle frailty; however, it cannot be excluded that there was an asymptomatic AWD pre-existing PD initiation.

Our data strengthen the notion that a one-stage surgical procedure of simultaneous repair of AWD and peritoneal catheter insertion may offer clinical advantages to patients in some circumstances. In the case of late referral of a patient with advanced renal failure and concomitant presence of AWD, PD treatment may be initiated within a shorter time without the time-consuming double procedure of AWD repair and successive peritoneal catheter insertion. In addition, it may likely avoid the introduction of a central venous catheter for extracorporeal dialysis treatment, which could further postpone initiation of PD program.

It is worth noting that the prolonged follow-up of our study distinguishes it from others [9, 10]. In one study, 19 patients were followed up for a mean period of 22 months (range 6–48) [9], while in the other 21 patients had a mean follow-up of 24 months (range 6–39) [10].

In recent years, the insertion of peritoneal catheters, as also artero-venous fistula construction, has been personally managed by nephrologists. In the case of a patient with AWD, however, both nephrologist and surgeon must be present in the theatre during placement of the peritoneal catheter, as the nephrologist does not have the expertise required for AWD repair [11].

## Conclusions

The long-term peritoneal catheter survival and the absence of AWD recurrence during PD treatment found in our study suggest that simultaneous surgical AWD repair and peritoneal catheter insertion can be regarded as a safe surgical procedure. This strategy makes PD possible for some patients who would otherwise be excluded from the possibility of PD and, in addition, it eliminates the risks of repeated anaesthesia and reduces the costs of hospitalization.
